# The Migration and Deposition Behaviors of Montmorillonite and Kaolinite Particles in a Two-Dimensional Micromodel

**DOI:** 10.3390/ma15030855

**Published:** 2022-01-23

**Authors:** Bate Bate, Chao Chen, Pengfei Liu, Chen Zhou, Xiao Chen, Shaokai Nie, Kexin Chen, Yunmin Chen, Shuai Zhang

**Affiliations:** MOE Key Laboratory of Soft Soils and Geoenvironmental Engineering, Institute of Geotechnical Engineering, College of Civil Engineering and Architecture, Zhejiang University, Hangzhou 310058, China; batebate@zju.edu.cn (B.B.); chao_chen@zju.edu.cn (C.C.); hiyori@zju.edu.cn (P.L.); 112177@zju.edu.cn (C.Z.); 21712211@zju.edu.cn (X.C.); nsk@zju.edu.cn (S.N.); chenyunmin@zju.edu.cn (Y.C.)

**Keywords:** microfluidic model, bentonite aggregates, kaolinite aggregates, clogging, spatial distribution, salinity

## Abstract

The pick-up, migration, deposition, and clogging behaviors of fine particles are ubiquitous in many engineering applications, including contaminant remediation. Deposition and clogging are detrimental to the efficiency of environmental remediation, and their mechanisms are yet to be elucidated. Two-dimensional microfluidic models were developed to simulate the pore structure of porous media with unified particle sizes in this study. Kaolin and bentonite suspensions were introduced to microfluidic chips to observe their particle deposition and clogging behaviors. Interactions between interparticle forces and particle velocity profiles were investigated via computational fluid dynamics and discrete element method simulations. The results showed that (1) only the velocity vector toward the micropillars and drag forces in the reverse direction were prone to deposition; (2) due to the negligible weight of particles, the Stokes number implied that inertia was not the controlling factor causing deposition; and (3) the salinity of the carrying fluid increased the bentonite deposition because of the shrinkage of the diffused electrical double layer and an increase in aggregation force, whereas it had little effect on kaolin deposition.

## 1. Introduction

The pick-up, migration, deposition, and clogging of fine particles in porous media are frequently encountered phenomena in different engineering fields [1]. These applications include the injection of zero valence iron (ZVI) particles for environmental remediation [1,2,3,4], grouting [5], the piping failure of earthen dams [6], clogging and particle loss at the interface of the capillary break layer (coarse-grained layer) and moisture-retaining layer (fine-grained particles) in a cover system for blocking the ingress of oxygen and meteoric water into waste landfill [7], the clogging of sandstone in methane hydrate exploration [8], the movement of clay particles in rock and soil reducing the efficiency of oil recovery [9,10,11,12,13], the propping of fractures in hydraulic fracking [14,15], and the geological sequestration of CO_2_ [16,17]. ZVI particle injection has been proposed to remove heavy metals, organochlorine, and other contaminants in groundwater and soils [1,2,3,4]. However, its field application has been limited because of the short ZVI migration distance due to deposition and particle clogging, i.e., only a few meters from the injection point [18]. Kaolin and bentonite have been used to modify the surface charge and specific surface area of ZVI particles to increase their migration distance [19,20].

**Figure 1:**
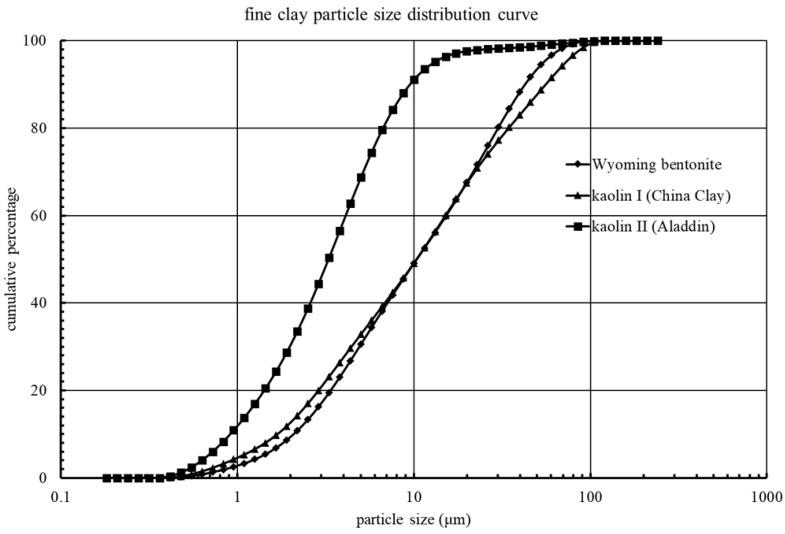
Particle size distribution curves of clay used in this study.

The known influencing factors of the aforementioned particle movement behaviors in porous media include the relative size of particles (compared to the pore throat geometry of the porous media), particle shape, surficial electrical properties of porous media, and the physicochemical properties of the carrying fluid [8,21]. The ratio of pore throat width to particle size (o/d) prompts the pore throat clogging phenomenon [21]. Particle blocking and bridging could occur at o/d ratios between 1.67 and 100 (Figure S1) [22,23]. Auset and Keller [24] reported the occurrence of pore throat clogging when o/d was less than 2.5 for a system with polystyrene latex particles (Figure 1). Cao et al. [8] used four types of particles (silica silt, bentonite, kaolin, and mica) mixed with deionized (DI) water and brine to find the critical o/d ratio in the range of 2.56–24. Platy-shaped particles have been demonstrated to clog more easily than spherical particles [25]. A summary of critical o/d ratios for clogging is shown in Figure S1.

High ion strength reduces the thickness of the diffused electric double layer and reduces the repulsive force among fine particles [26], resulting in fine clay particles depositing into and clogging the pore throats.

When gas and liquid phases coexist in a flow system in a porous medium, fine particles can be easily adsorbed, and they accumulate at gas–liquid interfaces [27,28,29]. Jung et al. [30] reported that fine particles in hydrate-bearing sediment could aggregate at the air–liquid interface after gas production, which in turn decreased gas production efficiency. Capillary energy potential and DLVO energy potential are key factors determining the fine particle behavior at air–liquid interfaces [29]. In non-flowing unsaturated porous media, particles would migrate toward interfaces to maintain a force balance [31,32] which could cause clogging at the pore throats. In a multiphase flowing system, however, the capillary energy potential of fine particles at air–liquid interfaces is several orders greater than the DLVO energy potential; thus, fine particles are easily mobilized by moving air–liquid interfaces [28].

Traditional particle migration experiments have been commonly conducted in soil columns [33,34] as field experiments [35] and numerical simulations [36,37]. However, such column tests limit the direct observation of real-time particle flow. On the other hand, microfluidic models have been widely used in the direct observation of multiphase flows in rock fissures [38], sandstone in methane hydrate exploration [8], microbial-induced carbonate precipitation in porous media [39], and enhanced oil recovery [40].

Previous studies have focused on the clogging threshold with little attention on pick-up and deposition behaviors, or fundamental mechanisms. The understanding of the mechanisms of the entire processes of the pick-up, migration, settlement, and deposition of fine particles in porous media would help predict and control the location and distribution of fine particles. Hence, the goal of this study was to simulate the migration and clogging behaviors of fine clay particles using a microfluidic model. The effects of different o/d ratios, grain size (D), position effect, and salinity of the carrying fluid were visually (by a micromodel) and numerically investigated.

## 2. Materials and Methods

### 2.1. Fine Clay Particle Materials

Wyoming bentonite (Wyo-Ben Inc., Billings, MT, USA), kaolin I (China Kaolin Clay Co., Ltd., Suzhou, China), and kaolin II (Aladdin, Shanghai, China) were used as fine particles in the experiment. Particle size distribution curves (Figure 1) were measured by a laser diffraction particle size analyzer (Mastersizer 2000, Malvern Instruments Ltd., Malvern, UK), with the mean particle sizes (d_50_) of bentonite, kaolin I, and kaolin II measured as 11.909, 8.962, and 3.283 μm, respectively. Clay particles were mixed with DI water and saturated NaCl solution with a particle concentration of 1 g/L. Clay suspension was driven into the microfluidic model using a peristaltic pump (Longer Precision Pump Co., Ltd., Baoding, China).

### 2.2. Micromodels

**Figure 2:**
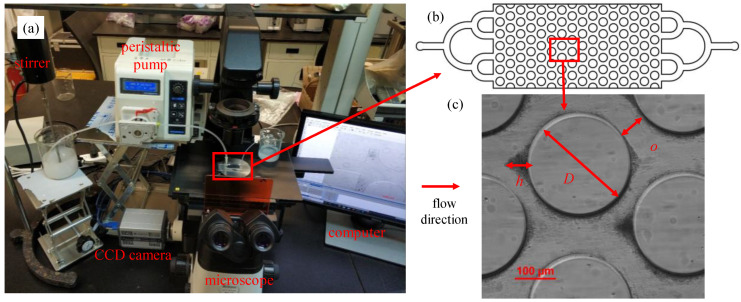
The illustration of microfluidic experimental devices: (**a**) composition of experimental equipment; (**b**) microfluidic chip; and (**c**) internal structure of microfluidic chip. D—micropillar diameter; o—pore throat width; h—deposition height.

Two-dimensional (2D) polydimethylsiloxane (PDMS) microfluidic chips (20 mm × 10 mm, length × width) (Figure 2b) were manufactured by the soft lithography technique. First, a silicon wafer and SU-8 photoresist were used to fabricate the mold with patterns designed to represent a simplified sand matrix (Figure 2c). Subsequently, liquid PDMS was poured over the mold. After solidification and hardening, PDMS was bonded to a glass slide by oxygen plasma [41]. The diameter of micropillars (D) was uniform in a single chip but varied across different chips (200, 250, 500, 1000, and 2000 μm, respectively). The pore throat width (o) of each chip was 20, 40, 60, 83, 100, 207, 414, and 830 μm, respectively. The micropillars measured 50 μm in height. The microfluidic chip patterns were centrosymmetric. Each chip had an inlet and outlet, both with binary tree-like structures, designed to ensure a uniform flow field.

### 2.3. Experimental Methods

The experimental setup is illustrated in Figure 2. A peristaltic pump was used to drive the suspension from the beaker into the microfluidic chip at a constant flow rate of 200 μL/min. A mechanical stirrer was adopted and operated throughout the experiment to prevent the settling of particles before pumping.

Microfluidic chips were cleaned using an ultrasonic cleaner with ethanol for 20 min, followed by DI water for another 20 min before each test. Before the clay suspension injection, DI water was pumped into the microfluidic chip to saturate the pore space. Due to the low permeability of PDMS, the trapped air could be discharged at a higher flow rate. The clay suspension was injected into microfluidic chips with various pore throat widths.

In addition, an inertial effect can be described by the Stokes number (Stk):

(1) $$S_{tk}=\frac{\rho_{p}d^{2}v}{18\mu D}$$

where *ρ_p_* is the particle mass density; *d* indicates the migration particle diameter; *v* denotes fluid velocity; *μ* is the fluid viscosity; and *D* is the micropillar diameter. A low Stokes number suggests that particles follow fluid streamlines (perfect advection), while a large Stokes number indicates the fact that particles are dominated by the inertia, and continue along their initial trajectory.

The flow of the clay suspension and deposition of clay particles were observed using an inverted microscope (Ti2-U, Nikon Corp., Tokyo, Japan) and recorded using a CMOS camera (Zyla 4.0, Andor Technology, Belfast, UK). Images were taken when the clay suspension flow reached a steady state. All experiments were conducted at room temperature (20 °C).

o/d ratios were 1.70 to 69.70 for bentonite, 2.23 to 92.61 for kaolin I, and 6.09 to 252.82 for kaolin II.

The pore fluid influences the electrical diffuse layer near the surface of fine particles; therefore, the stability of the suspension can be maintained by the combined effect of the van der Waals force between particles and the repulsive force of the diffused electric double layer (DLVO theory) [42,43]. In the DLVO theory, the thickness of the diffused electric double layer (θ) can be expressed by the following formula [44,45]:

(2) $$\theta =\sqrt{\frac{\varepsilon_{0}k}{2e_{0}^{2}N_{av}}}\cdot \sqrt{\frac{\kappa T}{cz^{2}}}$$

where *ε*_0_ = 8.85 × 10^−12^ F/m indicates the permittivity of free space, *k* = 1.38 × 10^−23^ J/K is the Boltzmann’s constant, *e*_0_ = 1.02 × 10^−19^ C denotes the electron charge, *N_av_* = 6.022 × 10^23^ mol^−1^ is Avogadro’s number, *κ* indicates the real permittivity of fluid (F/m), *T* represents the absolute temperature (K), *c* denotes the bulk fluid concentration (mol/L), and *z* represents the ionic valence.

### 2.4. Image Processing

An image analysis program written in Matlab R2018a was developed to quantify the height of clay particle depositions. The major processes were as follows. First, images were grayed, and appropriate thresholds were selected to ensure that the particle deposition profile in the binary image was consistent with that in the original image. The binary images were then processed by constructing a corrosion matrix to make the images only retain the binary image of particle deposition. Finally, the images were divided into blocks, a block matrix was assigned to each deposition, and the deposition area and height along the flow direction were calculated in pixels.

### 2.5. Numerical Simulation

**Figure 3:**
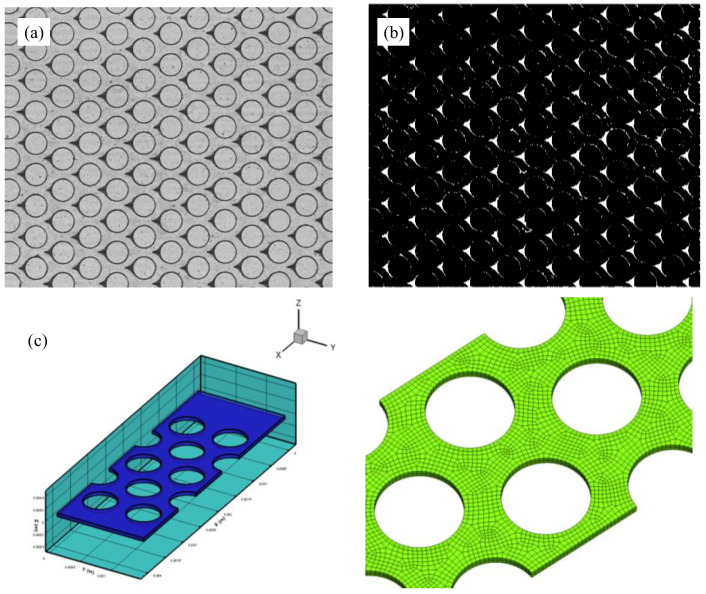
(**a**) The image captured by CMOS camera; (**b**) the image was binarized to calculate the number of pixels in front of the micropillars; and (**c**) simplified numerical simulation model.

Computational fluid dynamics (CFD) (Ansys Fluent) coupled with discrete element method (DEM) simulation, based on a Hertz–Mindlin with a Johnson–Kendall–Roberts (JKR) cohesion model [46] were developed to study the migration and deposition mechanism of fine particles in 2D microfluidic chips. The numerical simulation model is shown in Figure 3c.

The fluid phase was controlled by the Navier–Stokes equation and particles were governed by Newton’s second law:

(3) $$\rho \left(\frac{\partial V}{\partial t}+\left(V\cdot \nabla V\right)V\right)=f-\nabla P+\mu \nabla^{2}V$$

(4) $$m\frac{dv}{dt}=\sum_{i}^{}F_{i}$$

(5) $$I\frac{d\Omega }{dt}=\sum_{i}^{}M_{i}$$

where *ρ* represents the density of the fluid; *V* represents the velocity of the fluid; *t* represents time; *f* represents the external body force; *P* represents the static pressure; and *μ* represents the fluid viscosity. The contact among particles could be described by a Hertz–Mindlin with a JKR cohesion model:

(6) $$F_{JKR}=-4\sqrt{\pi \gamma E^{*}}\alpha^{\frac{3}{2}}+\frac{4E^{*}}{3R^{*}}\alpha^{3}$$

(7) $$\delta =\frac{\alpha^{2}}{R^{*}}-\sqrt{\frac{4\pi \gamma \alpha }{E^{*}}}$$

where *F_JKR_* represents the cohesion force, *γ* represents the surface energy density, *δ* represents the particle overlap distance, *α* represents the particle contact radius, E* represents the equivalent modulus, and R* represents the equivalent radius.

## 3. Results and Discussion

Deposition of clay particles was observed in front of the micropillars of the microfluidic chips after the clay suspension was pumped into the microfluidic chips. When the o/d was low, clogging also occurred at the pore throats. Four factors contributed to the deposition and clogging behaviors, namely the salinity of the carrying fluid, the specific o/d ratios, the micropillar diameter (D), and the distance along the flow direction.

### 3.1. Effect of Suspension Salinity

**Figure 4:**
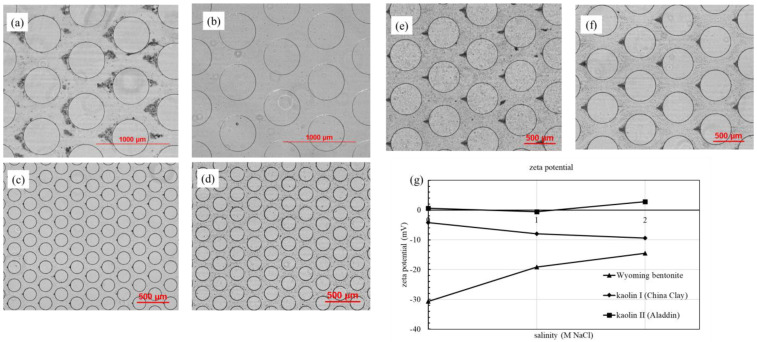
(**a**) 1 g/L Wyoming bentonite in saturated NaCl solution; (**b**) 1 g/L Wyoming bentonite in DI water; (**c**) Kaolin II in saturated NaCl solution; (**d**) Kaolin II in DI water; (**e**) Kaolin I in saturated NaCl solution; (**f**) Kaolin I in DI water; (**g**) Zeta potential values in different salinities. Flow direction: from left to right.

When the DI water was used as the carrying fluid, bentonite showed almost no deposition in front of the micropillars. When 1 mol/L or 2 mol/L of NaCl solution was used as the carrying fluid, the deposition of bentonite particles was significant (Figure 4a,b). At a constant flow rate of 200 μL/min in an NaCl solution and DI water, the bentonite particle migration experiment was repeated three times, and the accumulation height, volume and distribution were similar.

Three main forces control the stability of a clay suspension, namely van der Waals attraction, electrostatic repulsion, and the drag force caused by the flow [2].

When bentonite formed a suspension in DI water, the two forces could be balanced to form a substantially stable suspension. While bentonite was added to the brine, the high concentration of electrolyte compressed the thickness of the diffused electric double layer. As the electrostatic repulsion decreased, van der Waals forces became dominant, resulting in the deposition of particles inside the chips.

**Figure 5:**
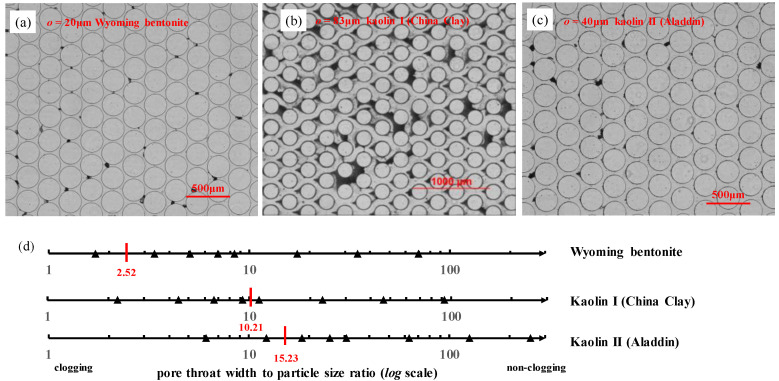
Images of fine clay particles with DI water clogging at pore throats in microfluidic chips: (**a**) Wyoming bentonite clogged at pore throats (o = 20 μm); (**b**) Kaolin I clogged at pore throats (o = 83 μm); (**c**) Kaolin II clogged at pore throats (o = 40 μm); and (**d**) the clogging and non-clogging boundaries of three kinds of fine clay particles.

The strength of repulsion or attraction between fine particles in the solution was measured by zeta (ζ) potential; the zeta potential values of clay suspension in different salinities are illustrated in Figure 5c. As observed, the absolute values for Wyoming bentonite decreased in value with the increasing salinity. The zeta potential of the initial suspension is −30.7 mV, implying that bentonite particles had good suspension stability in DI water. The zeta potential (in absolute value) decreased to −14.5 mV in 2_M NaCl solution; meanwhile, the bentonite suspension became unstable, and the bentonite particles were aggregated and easily deposited. Salinity, however, had little effect on the zeta potentials for kaolin I and kaolin II. This was consistent with the deposition behaviors of kaolin in the microfluidic chips, which did not change with salinity (Figure 4c,d).

The diffused electric double layer thickness between bentonite particles was compressed by NaCl, making it easier for the particles to be deposited and form aggregates, whereas NaCl had little effect on kaolin behavior. Bentonite particles require higher concentrations than those in brine to induce clogging in line with the Sogami-Ise model because bentonite particles do not readily cluster in the absence of positive ions in the water [47].

To explore the particle size variation range under different salinities, the average sizes of three kinds of clay were measured by Zetasizer Nano ZS90 (Malvern). The average sizes are listed in Table 1. The average size of Wyoming bentonite with DI water is approximately a quarter of that with 1 mol/L and 2 mol/L NaCl solution. The average size of kaolin I also increased with an increase in salinity, but kaolin II did not significantly change. This particle size variation is consistent with the deposition results shown in Figure 5. Notably, a certain difference between the average particle size data in Table 1 and the data in Figure 2 exists because different particle size measurement methods were used.

### 3.2. Particles Clogging at the Pore Throats

Local clogging was observed at certain o/d ratio values, however, the complete clogging of the entire porous media was not observed. The critical clogging o/d ratios of Wyoming bentonite, kaolin I, and kaolin II were observed to be 2.52, 10.21, and 15.23, respectively (Figure 5). Particles clogged at pore throats when the pore throat width was less than 20 μm for Wyoming bentonite (Figure 5a), 83 μm for kaolin I (Figure 5b), and 40 μm for kaolin II (Figure 5c). Analogously, at a constant flow rate of 50 μL/min and the given pore throat-to-fine particle size ratios (o/d = 2.6–36.4), 2% fine particles in DI water can migrate in the pore throat without bridging or clogging [48]. When o/d ratios were larger than the critical values, particle deposition in front of micropillars and non-clogging were observed. The critical clogging o/d ratios of kaolin in this study were similar to the results of Cao et al. [8], where such values for bentonite and kaolin (with a concentration of 0.1% w/w) of 13 and 12, respectively, were reported. The critical ratio difference of bentonite was because the particle size measured in our experiment (d50 = 11.909 μm) was significantly larger than the particle size used by Cao et al. [8] (d50 = 2 μm). With a high ratio of particle-to-pore throat size, low fines concentrations are required to induce clogging [47].

In addition, the attraction between clay particles and PDMS may lead to clay particles adsorbing onto the walls of the microfluidic system. The adsorbed particles narrow pore throats, increase o/d ratios, and make clogging occur easier than that to glass walls [21]. Similarly, it was reported that latex particles could be attracted by PDMS walls [22].

### 3.3. Particle Deposition Criteria

**Figure 6:**
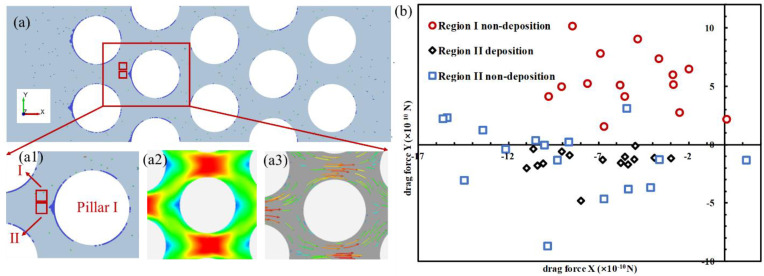
(**a**) Particle drag force X and drag force Y in Region I and Region II as recorded, and the contour and vector map of fluid velocity; (**b**) distribution of drag force X and drag force Y of the deposited and non-deposited particles in Region I and Region II.

Deposition in front of micropillars could happen when particles migrated in a microfluidic chip. CFD-DEM simulation results (Figure 6) revealed the particle deposition process (Figure 6(a1)), as well as the contour of velocity magnitude (Figure 6(a2)) and the velocity vector field (Figure 6(a3)). Two regions of identical shape and size were chosen in front of a pillar (Pillar I) for force analysis: Region I was located in the front of Pillar I, whereas Region II was above Region I (Figure 6(a1)). Particle trajectories were governed by acting forces, including hydrodynamic force, interparticle electrical force, and gravitational force. Given the sparse distribution of particles in the running fluid in Regions I and II, the electrical force did not contribute unless particles were in close affinity (as in the deposited region). The direction of gravity was perpendicular to the microfluidic chip plane, which did not contribute to the deposition behavior.

In this experiment, the calculated Stokes numbers were significantly less than unity, which meant that the movement of clay particles was mainly controlled by fluid dynamics, and the inertia of particles had little effect on their trajectories. Therefore, the hydrodynamic force is the main contributor to particle deposition behaviors [6].

The hydrodynamic force induced by the flow of fluid on migrating particles, also known as a drag force, was calculated (Equations (8)–(10)) and presented in the X and Y directions (Figure 6b). Two observations were made: (1) only particles passing through Region II were deposited; (2) in Region II, particles with a small force in the Y direction were statistically more prone to deposition than those with a large force in the Y direction. The primary role of drag forces in Region II was decelerating particles, whose velocity vector direction was primarily horizontal. The small force in the Y direction made particles unlikely to travel around the pillars, facilitating deposition:

(8) $$\frac{du_{p}}{dt}=F_{D}\left(u-u_{p}\right)+\frac{g_{x}\left(\rho_{p}-\rho \right)}{\rho_{p}}+F_{x}$$

(9) $$Re=\frac{\rho d_{p}\left|u_{p}-u\right|}{\mu }$$

(10) $$F_{D}=\frac{18\mu }{\rho_{p}d_{p}^{2}}\frac{C_{D}Re}{24}$$

where *F_D_* (*u − u_p_*) is the drag force per unit particles mass, *u_p_* is particle velocity, *u* is fluid velocity.

### 3.4. Effects of Micropillar Diameter on Particle Deposition

**Figure 7:**
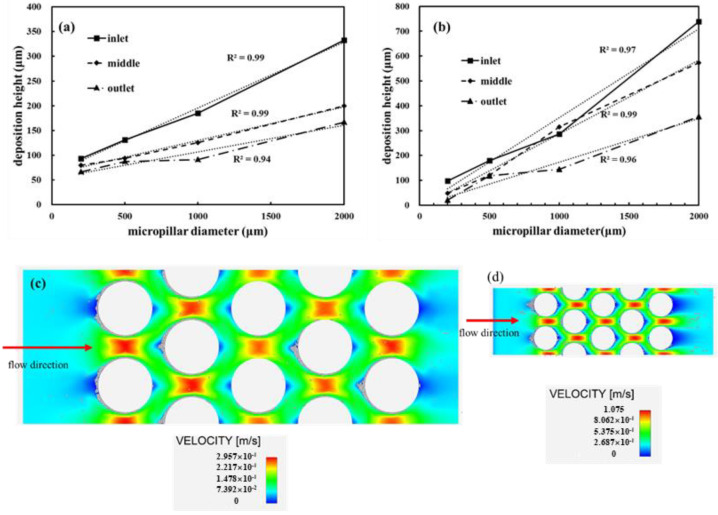
The deposition height of clay particles increased with micropillar diameter: (**a**) 1/L kaolin I in DI water; (**b**) 1 g/L Wyoming bentonite in saturated NaCl solution. Velocity distribution diagram of the microfluidic chips with micropillar diameters of (**c**) 500 μm and (**d**) 200 μm.

The particle deposition height of kaolin I in DI water and bentonite in saturated NaCl solution under different micropillar diameters is illustrated in Figure 7. An approximately linear relationship between the deposition height and micropillar diameter was observed.

For kaolin I in DI water, the particle deposition height increased from 93 to 333 μm at the inlet, from 79 to 200 μm at the middle part, and from 66 to 166 μm at the outlet (Figure 7a), respectively. A similar trend was observed for bentonite, with an increase in deposition height from 98 to 738 μm at the inlet, from 48 to 573 μm at the middle part, and from 20 to 356 μm at the outlet (Figure 7b).

The shapes of the velocity contours of the simulated chips at different micropillar diameters were similar (Figure 7c,d). Triangular regions in front of the micropillars exhibited low velocity (approximately 70% of the inlet velocity) and hosted the majority of deposited particles. As the diameter of the micropillar increased, the low-velocity region increased nearly proportionally. This could lead to the aforementioned trend of a linear increment in the deposition height with the micropillar diameter.

At low flow rates, particles can only stick to the wall outside the exclusion zone around each deposited particle. This repulsion prevents the formation of aggregates. At sufficiently large velocities, repulsive forces between particles can be overcome and multilayer clusters form, resulting in the growth of aggregates [49].

### 3.5. Deposition Height Decline along the Flow Direction

**Figure 8:**
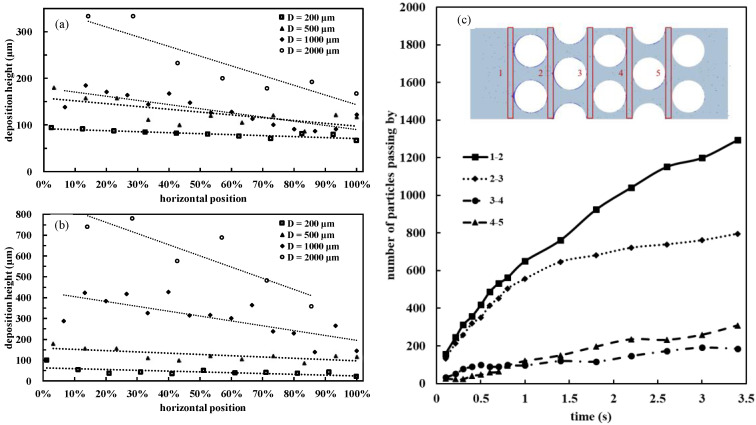
The measured deposition height in front of micropillars decreased from the inlet to the outlet of the microfluidic chips: (**a**) 1 g/L kaolin I in DI water; and (**b**) 1 g/L Wyoming bentonite in a saturated NaCl solution. The distance from the first pillar to the last pillar was normalized by the total distance (i.e., a percentage value). The numerical simulation of a five-column chip: (**c**) deposited particles in areas 1–5, and the reduced number of particles between two adjacent areas.

The spatial distribution of the particle deposition height of kaolin I in DI water and bentonite in saturated NaCl solution with various micropillar diameters (200, 500, 1000, and 2000 μm) is plotted in Figure 8. The height of the deposition in front of the pillars in microfluidic chips decreased along the flow direction (Figure 8a,b). For the chip with a micropillar diameter of 2000 μm, the deposition height decreased to 50% of the deposition height of the first column, whereas the deposition height decreased to 70% of the first column for the chip with a 200 μm micropillar diameter. The lower deposition height percentage for the 2000 μm diameter chip was probably because the flow system had not reached equilibrium at the limited experimental duration. There was an approximately linear relationship between the deposition height and micropillar diameter [47]: as the diameter of the micropillar increased, the deposition height increased. Given the fact that the available particles along the flow direction decreased, the reduction in the deposition height of particles seemed reasonable. However, the ratio of reduction between two adjacent columns along the flow direction was not constant, as illustrated in a numerical simulation (Figure 8c). The number of particles deposited between recording areas 1 and 2 (i.e., lines 1–2 in Figure 8c) increased the fastest, and the total number of particles deposited was the largest. Simulation results were consistent with the experimental results in that the number of particles deposited in the front of the micropillars decreased along the flow direction. Naturally, the particle concentrations near the injection point are ubiquitous, which was also observed in this study.

### 3.6. Effect of Air–Liquid Two-Phase Flow

**Figure 9:**
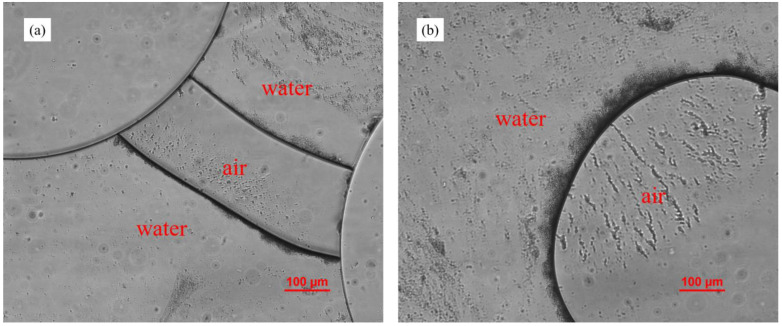
(**a**) Kaolin I particles aggregated at air–liquid interfaces, (**b**) zoomed-in view of theair-water interface.

In methane hydrate exploration, ZVI injection, and other engineering fields, the presence of a gas phase plays a significant role in engineering efficiency. Consequently, it is important to investigate how gases influence fine particle distribution in porous media. In this study, air was injected into the microfluidic chips in which a stable deposition of kaolin I particles had formed. After introducing air into these chips, several water–air interfaces were formed (Figure 9). It was also observed that kaolin particles concentrated at these interfaces, and clogging easily occurred at the pore throats.

Particles tend to be adsorbed to air–liquid interfaces in a multiphase system. The migration of fine particles along the water–gas interface locally increased fine particle concentration and subsequently caused clogging in the pore throat [48]. Self-driven capillary flow pushes suspended particles to the droplet edge in a coffee-ring effect [50]. As a non-wetting phase (air) invading porous media, fine particles migrate to and aggregate at the interfaces [31]. In a quiescent system, the particles are activated by Brownian motion toward air–liquid interfaces and are trapped by capillary forces [32]. Gravity, buoyancy, air–liquid, air–solid, and liquid–solid interfacial tensions create a force balance of particles already present at air–liquid interfaces [31]. In our experiment, fine particles concentrated at air–liquid interfaces, which caused pore throat clogging and blockage. However, the increasing flow rate caused air–liquid interfaces to move on, the triangular aggregates in front of the micropillars to rapidly change, and most of the aggregates to be washed away.

### 3.7. Applicability in Related Engineering Field

Fines transportation in geomaterials is one of the fundamental phenomena in nature and in engineering practices, such as soil erosion and scour, the piping failure of a dam, the grouting of bentonite slurry, zero-valence ion (ZVI) injection, clogging during methane hydrate exploration, clay migration in enhanced oil recovery, and proppant or gel injection in hydraulic fracturing. Much attention was given to clogging or fines loss behavior, while the study of the deposition of fines onto the porous media, especially at a particulate level, is still lacking. This study filled in the gap and identified the factors influencing the deposition behavior. This study is applicable to the field of grouting as well as the co-transportation of ZVI and clay particles in contaminated site remediation, where the suspension properties, velocity, and salinity are crucial to the uniformity and transport distance. This study was also applicable to the fines migration associated with methane hydrate exploration and enhanced oil recovery. When the size of the fines (either natural clay particles or artificially manufactured proppants or gel) is at least 10 times smaller than the pore-throat size, deposition is the major phenomenon for fines retardation in a porous media.

## 4. Conclusions

2D microfluidic models were developed to evaluate the fine kaolin and bentonite particle migration and clogging behaviors in porous media. The microfluidic models were fabricated using the soft lithography method, and the pore network structure consisted of staggered micropillars and pore throats. DI water and a NaCl solution were used as carrying fluids to transport fine clay particles.

The o/d ratio was the main factor that controlled the fine clogging behavior at pore throats. The critical clogging o/d ratios of Wyoming bentonite, kaolin I, and kaolin II were 2.52, 10.21, and 15.23, respectively. Clay particles could smoothly pass through the pore structure in microfluidic chips, when the ratio was larger than the critical value, and neither bridging nor clogging was observed.

However, particles were deposited in front of the micropillars and formed triangle-like depositions when no clogging occurred. The front part of the larger micropillar formed depositions with a larger area and height, which was related to the flow field inside the chip. The hydrodynamic force was the main factor that controlled particle deposition in front of micropillars. A smaller value of drag force Y caused the particles to deposit. When the direction of the streamline changed, clay particles deviated from the streamline under the action of inertia and then entered the triangle-like low-velocity area in front of the micropillars. Larger micropillars formed a wider range of low-velocity areas, resulting in larger-sized depositions. In addition, the height of the deposition gradually decreased with the flow direction, which was consistent with numerical simulation results.

The ionic strength of the carrying fluid affected the electrical interaction between clay particles. Specifically, the rise in salinity broke the balance of diffused electric double layer force and van der Waals forces between bentonite particles in the suspension and enhanced the bentonite deposition effect. However, salinity had almost no effect on kaolin deposition in the microfluidic chips. In addition, the strong ability of air–liquid interfaces in the flow system to collect fine particles could increase the possibility of particles clogging the pore throats.

## Figures and Tables

**Table 1:** Average size of Wyoming bentonite, kaolin I (China Cay), and kaolin II (Aladdin) particles with DI water, 1 mol/L, and 2 mol/L NaCl solution.

	Clay	Wyoming Bentonite	Kaolin I (China Cay)	Kaolin II (Aladdin)
Carrying Fluid				
DI water		1.16 μm	1.28 μm	0.92 μm
1 mol/L NaCl		4.31 μm	2.55 μm	1.35 μm
2 mol/L NaCl		4.11 μm	2.30 μm	1.05 μm

## Data Availability

The data presented in this study are available in Supplementary Materials.

## Supplementary Materials

The following are available online at https://www.mdpi.com/article/10.3390/ma15030855/s1, Figure S1: A summary of o/d values critical for clogging.
