# Supplementary material for: The Migration and Deposition Behaviors of Montmorillonite and Kaolinite Particles in a Two-Dimensional Micromodel
Source: Materials (Basel). 2022 Jan 23;15(3):855. doi: 10.3390/ma15030855 (PMC8838163; doi:10.3390/ma15030855)
Supplement: Supplementary file 1 [file materials-15-00855-s001.zip › materials-1442188-supplementary.pdf]

materials

MDPI

Supplementary Materials

The Migration and Deposition Behaviors of Montmorillonite and Kaolinite Particles in a Two-Dimensional Micromodel

Bate Bate [1], Chao Chen [1], Pengfei Liu [1], Chen Zhou [1], Xiao Chen [1], Shaokai Nie [1], Kexin Chen [1], Yunmin Chen [1] and Shuai Zhang [1,*]

[1] MOE Key Laboratory of Soft Soils and Geoenvironmental Engineering, Institute of Geotechnical Engineering, College of Civil Engineering and Architecture, Zhejiang University, 310058, Hangzhou, China; batebate@zju.edu.cn (B.B.); chao_chen@zju.edu.cn (C.C.); hiyori@zju.edu.cn (P.L.); 112177@zju.edu.cn (C.Z.); 21712211@zju.edu.cn (X.C.); nsk@zju.edu.cn (S.N.); chenkexin@zju.edu.cn (K.C.); chenyunmin@zju.edu.cn (Y.C.)

* Correspondence: zhangshuaiqj@zju.edu.cn

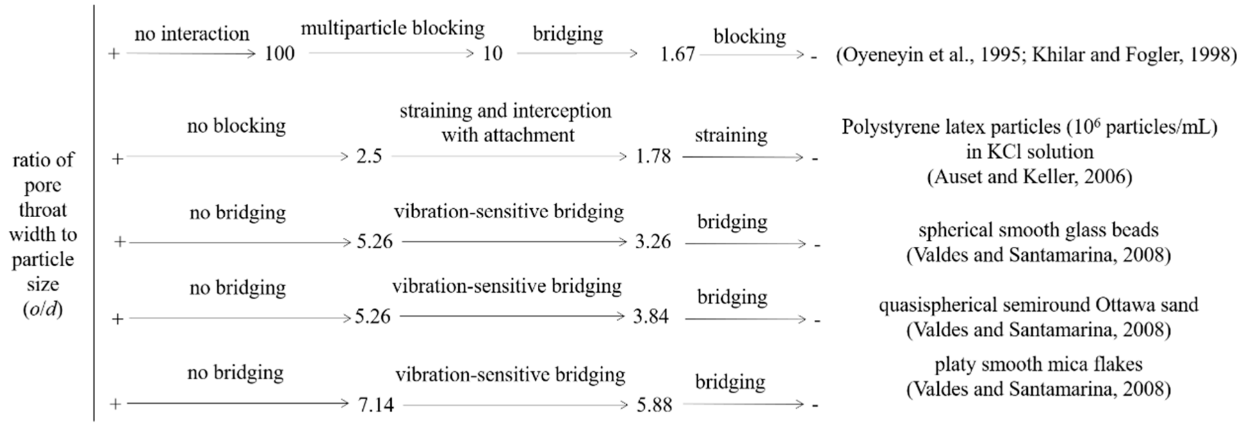

Figure S1. A summary of o/d values critical for clogging.
